# S100B in brain–gut–liver crosstalk: from glial activation to multiorgan inflammation

**DOI:** 10.3389/fimmu.2026.1933882

**Published:** 2026-09-17

**Authors:** Qi Liu, Yan Ming, Chunlei Ji, Lian Fu, Ruohua Li, Zhengyi Yang, Sha Wen

**Affiliations:** 1 Department of Gastroenterology, Zhejiang Provincial People’s Hospital Bijie Hospital (The First People’s Hospital of Bijie City), Bijie, China; 2 Department of Neurology, Zhejiang Provincial People’s Hospital Bijie Hospital (The First People’s Hospital of Bijie City), Bijie, China

**Keywords:** brain-gut-liver axis, enteric glia, gut microbiota, hepatic encephalopathy, intestinal barrier, liver fibrosis, neuroinflammation, S100B

## Abstract

S100 calcium-binding protein B (S100B) is conventionally viewed as a marker of astrocytic injury, blood–brain barrier (BBB) disruption, and neurological disorders. Emerging evidence places S100B within the broader brain–gut–liver axis of inflammatory signaling. This review proposes that S100B is a glial-derived integrating effector that couples enteric glial activation, barrier dysfunction, hepatic fibrosis, and hepatic encephalopathy (HE) into a multi-organ inflammatory network. Its spatial expression map, release kinetics, alarmin (damage-associated molecular pattern, DAMP) activity, and immune-cell infiltration differ among the gut, liver, and brain, shaping axis signaling. S100B is predominantly expressed in astrocytes of the central nervous system, Schwann cells, and enteric glial cells and is also detected in liver tumor immune cells, biliary epithelial cells, and activated hepatic stellate cell (HSC)-associated lesions. It exerts context-dependent functions: at low, nanomolar concentrations it supports neurotrophic activity and barrier integrity, whereas sustained elevation acts as a DAMP that amplifies inflammatory responses through receptor for advanced glycation end products (RAGE), Toll-like receptor 2 (TLR2)/Toll-like receptor 4 (TLR4), nuclear factor kappa B (NF-κB), NOD-, LRR-, and pyrin domain-containing protein 3 (NLRP3) and a disintegrin and metalloproteinase 10 (ADAM10)/β-catenin pathways. Clinically, S100B kinetics, a rapid release with a short half-life after acute injury versus chronic multi-organ spillover in cirrhosis, determines whether elevation reflects a local glial event or systemic disease burden. S100B has been implicated in enteric infectious enteritis, inflammatory bowel disease (IBD), diarrhea-predominant irritable bowel syndrome (IBS-D), cholestatic fibrosis, metabolic dysfunction-associated steatotic liver disease (MASLD), alcohol-associated liver disease (ALD), viral hepatitis, hepatocellular carcinoma (HCC), and HE with comorbid neuroinflammation. Available evidence suggests that S100B is a candidate link between glial responses, barrier dysfunction, hepatic inflammation, and neuroinflammation within the brain–gut–liver axis, although its causal role, tissue origin, and clinical utility require further validation.

## 1 Introduction

The brain–gut–liver axis is a multisystem regulatory network comprising the central nervous system, enteric nervous system, vagus nerve, gut microbiota, intestinal barrier, portal circulation, bile acid metabolism, and hepatic immune–metabolic system (1). Historically, the gut–liver axis has been used primarily to explain how dysbiosis, intestinal leakage, and portal endotoxins drive hepatic inflammation (2, 3), while the gut–brain axis emphasizes the impact of microbial metabolites, vagal signals, and neuroimmune crosstalk on cognition, mood, and neuroinflammation (4–6). With growing insights into hepatic encephalopathy (HE), alcohol-associated liver disease (ALD), metabolic dysfunction-associated steatotic liver disease (MASLD), and neurodegenerative disorders, the interplay between the intestine, liver, and brain can no longer be treated as two parallel axes. It is more appropriately viewed as a multi-organ network with positive feedback amplification (7–10) (**Figure 1**).

**Figure 1:**
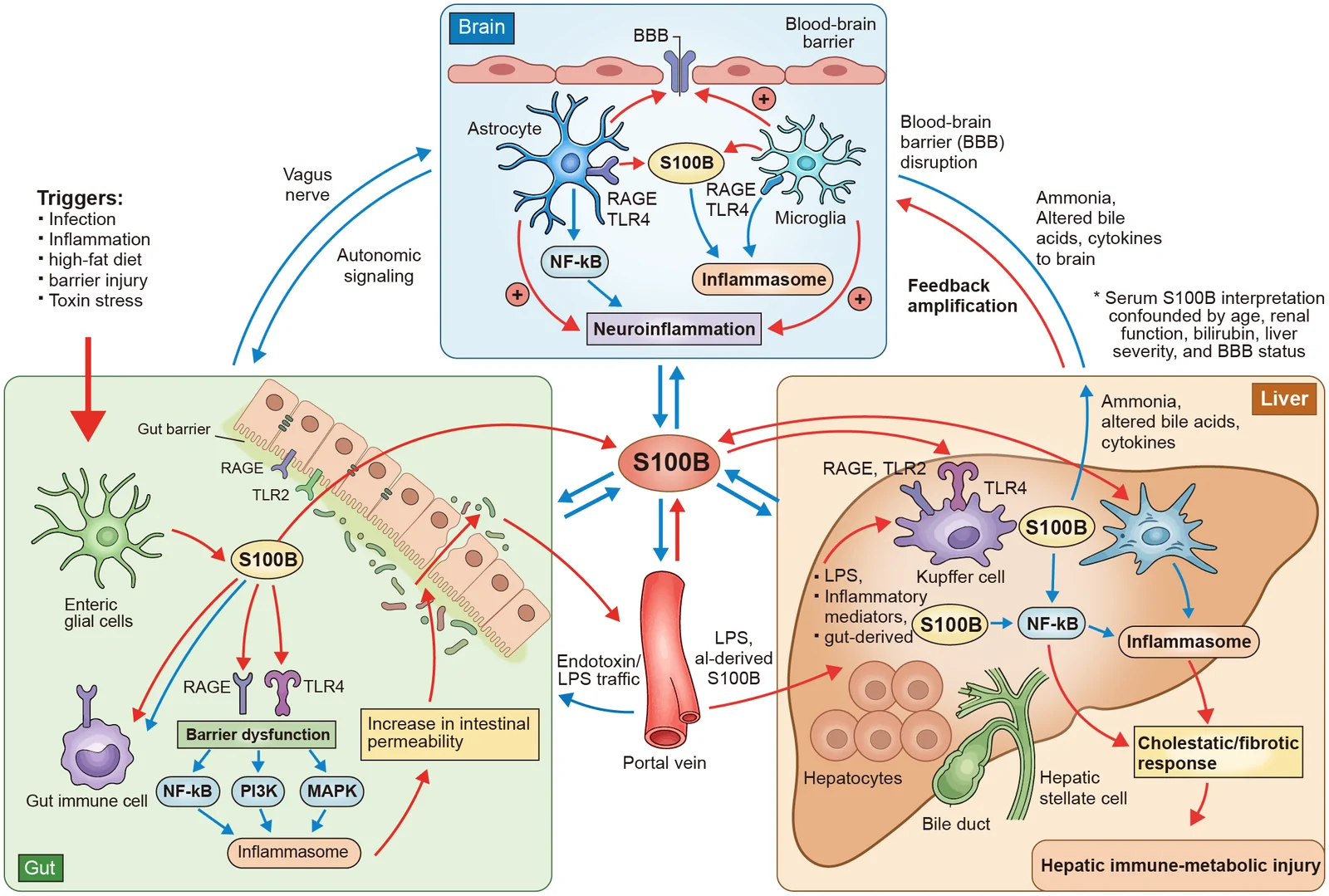
S100B-centered brain-gut-liver axis. Pathogenic factors (infection, HFD, etc.) activate EGCs, which release S100B, thereby disrupting the intestinal barrier via the RAGE/TLR4 and NF-κB/inflammasome pathways. Intestinal LPS, S100B, and other mediators are transported via the portal vein to the liver, where they activate Kupffer cells and HSCs, triggering hepatic immune-metabolic injury and ductular fibrotic responses. The damaged liver releases ammonia, bile acids and cytokines that compromise the BBB, subsequently activating brain astrocytes and microglia to release more S100B and exacerbate neuroinflammation. The intestine, liver and brain communicate through both vagal and hematogenous routes, forming a bidirectional vicious cycle.

S100 calcium-binding protein B (S100B) is a Ca²^+^-binding protein of the S100 family and is thought to originate mainly from astrocytes and Schwann cells (11). Recent studies have identified enteric glial cells (EGCs) as another source of S100B triggered by infection, inflammation, high-fat diet (HFD), barrier injury, or toxin exposure (12–14). Intracellular S100B participates in cytoskeletal dynamics, Ca²^+^ homeostasis and cellular repair (15, 16); extracellular S100B engages receptor for advanced glycation end products (RAGE), Toll-like receptor 2 (TLR2) and Toll-like receptor 4 (TLR4) to activate nuclear factor kappa B (NF-κB), phosphoinositide 3-kinase (PI3K), mitogen-activated protein kinase (MAPK) and inflammasome signaling, exhibiting DAMP-like inflammatory activity (17–20) (**Figure 1**).

This dual nature makes S100B a candidate for understanding pathological transitions in the brain–gut–liver axis. In the gut, EGC-derived S100B may affect epithelial tight junctions, vascular barrier integrity, and mucosal immunity (21–23); in the liver, the S100B/RAGE axis has been implicated in bile duct ligation-induced cholestatic fibrosis (24), while in the brain, S100B participates in astrocytic responses, blood–brain barrier (BBB) dysfunction, and neuroinflammation in HE (24, 25) (**Figure 1**). However, the interpretation of serum S100B levels in humans is complicated by confounding factors, including age, renal function, bilirubin level, liver disease severity, extracerebral sources, and BBB integrity (25, 26). Why then focus on the entire review of a single protein? These three properties justify our choice. First, S100B is among the most abundant soluble proteins of the nervous system and serves as a clinically measurable biomarker (e.g., in traumatic brain injury and melanoma follow-up); therefore, any additional biological insight carries immediate translational relevance (26–28). Second, S100B is expressed and dynamically regulated in all three organs of the axis: glia of the central and peripheral nervous systems, enteric glia in the gut, cholangiocytes, and activated hepatic stellate cells (HSCs) in the injured liver, a distribution that is unusual among S100 proteins that allows S100B to couple to distant compartments (11, 21, 24). Third, S100B sits at the interface between trophic (repair-promoting) and pathological (DAMP-like) functions, so its net effect depends on release kinetics, local concentration, receptor profile, and disease stage; clarifying these determinants may offer a tractable, stage-specific window for intervention (28, 29). These considerations, detailed throughout this review, explain why S100B, rather than another member of the S100 family, was the focal molecule of this study.

## 2 S100B biology in the brain–gut–liver axis

The actions of S100B in this axis are context-dependent rather than simply detrimental or beneficial. A more nuanced view is that its effects depend on the site of release, local concentration, receptor expression profile, context of tissue injury, and clearance capacity (**Table 1**). S100B can support neuronal survival, glial maturation, synaptic maintenance, tissue repair and barrier homeostasis (30); under inflammatory, ischemic, toxic, dysbiotic or hyperammonemic conditions, sustained S100B release may convert it into an inflammatory amplifier (19, 26).

**Table 1:** S100B in the brain-gut-liver axis: cellular sources, pathways, and functional implications.

Axis compartment	Major cellular source	Main pathways	Physiological role	Pathological implication	Data type (preclinical/clinical)	References
Brain and Peripheral Nervous system	Astrocytes, Schwann cells, glial-lineage cells	RAGE, TLR2, vascular endothelial growth factor (VEGF)/vascular endothelial growth factor receptor 2 (VEGFR2), cyclooxygenase-2 (COX-2), NF-κB, oxidative stress	Neurotrophic support, synaptic and metabolic regulation	Neuroinflammation, BBB dysfunction, oxidative injury, cognitive impairment	Preclinical + clinical (serum/cerebrospinal fluid (CSF))	(17, 18, 33)
Gut	Enteric glial cells	RAGE/PI3K/NF-κB, TLR4, NOD-, LRR-, and pyrin domain-containing protein 3 (NLRP3), aryl hydrocarbon receptor (AHR)/indole pathway, ADAM10/β-catenin	Motility regulation, epithelial support, neuroimmune sensing	Barrier disruption, interleukin-6 (IL-6)/tumor necrosis factor alpha (TNF-α)/interleukin-1 beta (IL-1β) release, visceral pain, colitis amplification	Preclinical + human mucosal/serum studies	(12–14, 36)
Intestinal barrier and gut vascular barrier (GVB)	Enteric glia-epithelial-endothelial interface	Tight junction proteins, β-catenin, caspase-8, ADAM10	Maintains epithelial and vascular selectivity	Portal endotoxemia, microbial translocation, liver inflammation	Preclinical	(14, 46)
Liver	Cholangiocytes, ductular reaction areas, activated HSC-associated lesions	S100B/RAGE, α-SMA, Col1a1, TIMP-1	Limited expression under basal conditions	Cholestatic ductular reaction and fibrosis	Preclinical + bioinformatics (HCC)	(24)
Circulation	Composite release from brain, gut, liver, adipose, and other extracerebral sources	Biomarker rather than a pathway-specific readout	Potential systemic injury signal	Low organ specificity; influenced by age, renal function, bilirubin, and Model for End-Stage Liver Disease (MELD)	Clinical	(25–27)

RAGE is one of the best-characterized receptors of S100B. It is a multi-ligand pattern-recognition receptor that binds S100B, high-mobility group box 1 (HMGB1), advanced glycation end products (AGEs) and other DAMPs, and signals through NF-κB, MAPK, c-Jun N-terminal kinase (JNK), extracellular signal-regulated kinase (ERK) and oxidative stress pathways (31, 32). In the nervous system, S100B/RAGE signaling is linked to astrocytic reactivity, endothelial injury, neutrophil extracellular trap (NET) formation, and neuroinflammation (18, 33, 34). In the bile-duct-ligation model, S100B and RAGE are co-upregulated in biliary epithelial cells and activated HSCs; recombinant S100B promoted alpha-smooth muscle actin (α-SMA) and Col1a1 expression in HSC-T6 cells, whereas RAGE siRNA attenuated this effect (24).

Notably, not all S100B signaling is RAGE-dependent. Studies on enteric glia have indicated that S100B contributes to colonic motility and cholinergic excitability through mechanisms that may be independent of RAGE (35), underscoring the context dependence of S100B actions. Similarly, S100B can signal through the a disintegrin and metalloproteinase 10 (ADAM10)/β-catenin axis and through interactions with growth factors in non-neuronal and intestinal contexts, further expanding the portfolio of RAGE-independent pathways (36, 37).

## 3 S100B ontogeny follows a stereotypic developmental trajectory

S100B is an early marker of astroglial commitment that is detectable in neural-crest-derived structures and radial glia during embryogenesis, increases with astrocytic maturation, and reaches maximal levels in the adult brain (16, 28). Circulating levels mirror this trajectory (relatively high at birth and in early infancy, declining to picomolar values around puberty, and rising again with aging-related astrocytic reactivity) (29, 38). In the gut, EGCs are derived from the neural crest, and S100B immunoreactivity appears as the enteric nervous system matures, serving as a routine EGC marker (22, 39). As S100B is trophic during development but can acquire damage-associated molecular pattern (DAMP) functions with age or pathology, age-dependent reference ranges are required in hepatology and geriatric cohorts, and developmental stage and biological age should be recorded in studies relating S100B to organ dysfunction (16, 29).

## 4 S100B is expressed, released and regulated in a tightly controlled manner in adults

S100B is a small (~21 kDa, 92-amino-acid) homodimeric Ca2+-binding protein of the EF-hand type, expressed in adults mainly by glial lineage cells, including mature and reactive astrocytes, Schwann cells, satellite glial cells, and enteric glial cells in the gut (11, 28). Astrocytes constitutively secrete S100B, and at nanomolar concentrations, they exert neurotrophic and gliotrophic effects (28, 29); their release increases further after cell injury, infection, inflammation, hyperammonemia, hypoxia, and BBB disruption (16, 17, 29). Systemic levels are shaped by age, sex, adipocyte-derived extracerebral fraction, and renal clearance. With a blood half-life of only ~25 min, steady-state serum S100B reflects the production–elimination balance rather than pure astrocytic injury (26, 40). Along this axis, the EGC-derived release is triggered by microbial products, toxins, HFD, and barrier injury, whereas astrocytic S100B release is induced by ammonia, cytokines, and oxidative stress (12–14, 17). Microbiota metabolites exert opposing effects; butyrate suppresses EGC S100B, whereas the loss of indole–AHR signaling relieves tonic restraint and permits S100B/NLRP3 activation (14, 36). Thus, S100B is a sensitive but multi-determinant readout of glial and barrier statuses.

## 5 S100B displays a distinct spatial expression map across the brain–gut–liver axis

S100B distribution differed markedly across the axes (**Table 2**). In the brain, it is expressed at high levels by astrocytes in the cortex, hippocampus, and white matter, as well as by Schwann cells; CSF and serum reflect both cellular sources. In the gut, EGCs of the myenteric and submucosal plexuses and mucosal lamina propria produce S100B adjacent to the epithelium and vessels. In healthy livers, basal S100B expression is low or undetectable, but is induced in cholangiocytes, ductular-reaction areas, and activated HSCs upon cholestatic or fibrotic injury (24). In HCC, by contrast, transcriptomic deconvolution attributed S100B predominantly to tumor-infiltrating immune cells (natural killer and Kupffer cells) rather than to malignant hepatocytes, with expression correlating with immune cell infiltration (41). Such cell type-specific, spatially resolved patterns indicate that single-cell and spatial transcriptomics, along with multiplexed immunohistochemistry, are required to attribute an S100B alteration to its correct cellular source before targeting (**Table 2**).

**Table 2:** Spatial expression map, reported S100B levels and kinetics across the brain–gut–liver axis.

Organ/ compartment	Predominant S100B-positive cell types	Basal abundance	Changes during dysfunction and kinetics	Cross-organ (distant) relevance	Evidence type	References
Brain (CNS)	Mature and reactive astrocytes; oligodendrocyte-lineage glia	High; constitutive secretion; reflected in CSF/serum	Acute injury: rapid release, serum peak, short half-life (≈25 min); chronic: accumulation with gliosis and BBB disruption	Systemic spillover in cirrhosis raises serum S100B; astroglial loop sustains HE neuroinflammation	Preclinical + clinical	(16, 26, 29, 40)
Peripheral nervous system	Schwann cells, satellite glial cells	Moderate–high	Nerve injury: upregulation during degeneration/regeneration	Peripheral and enteric glial responses run in parallel (e.g., IBD, visceral pain)	Preclinical	(11, 28, 29)
Gut	Enteric glial cells (myenteric and submucosal plexuses; mucosal lamina propria)	Moderate (EGC marker)	Infection, IBD/ulcerative colitis (UC), 5-fluorouracil (5-FU), HFD: upregulation; release triggered by toxins and microbial metabolites	EGC S100B weakens epithelial/vascular barriers and raises portal inflammatory traffic to the liver	Preclinical + clinical	(12–14, 36, 46)
Liver	Cholangiocytes, ductular-reaction cells, activated HSCs; immune cells (NK, Kupffer) in HCC	Low/absent basally	Cholestasis (BDL): induced with ductular reaction and fibrosis progression; serum rises with MELD in cirrhosis	Fibrotic/cirrhotic liver generates systemic S100B and inflammatory mediators that reach the brain (HE)	Preclinical + bioinformatics	(24, 25, 41)
Circulation	Composite (glial, gut, adipose, liver-derived)	Serum ≈36–44 pg/mL in cirrhosis; healthy reference ≈15.6 ng/L (commercial assay)	Biological half-life ≈25 min; acute vs. chronic kinetic profiles distinguish local vs. systemic burden	Integrates organ inputs; requires age/renal-function adjustment and kinetic sampling	Clinical	(25, 40, 43)

## 6 S100B acts as an alarmin and sustains microenvironmental crosstalk in all three tissues

Extracellular S100B fulfills the operational criteria of a damage-associated molecular pattern (DAMP) or alarmin: released from stressed or damaged cells, it engages RAGE, TLR2 and TLR4 on tissue-resident and innate immune cells and triggers pro-inflammatory programs (29, 42). Unlike the phagocyte-restricted alarmins S100A8/A9, S100B is constitutively secreted by glia; therefore, sustained glial injury converts a homeostatic intercellular signal into an inflammatory amplifier (29, 42). This alarmin activity sustains bidirectional glial–microenvironment crosstalk in each tissue: astrocyte-derived S100B activates microglia and the endothelium, promotes NET formation, and reinforces reactive gliosis (18, 29); EGC-derived S100B amplifies mucosal cytokine release and compromises tight-junction proteins (12, 13, 21); and cholangiocyte/HSC-derived S100B engages RAGE to promote ductular reactions and fibrogenesis (24). As S100B also upregulates RAGE in target cells, a feed-forward loop can propagate inflammation from one organ to the next and is central to a coordinated gut–liver–brain inflammatory network (29).

## 7 S100B kinetics shift with disease progression and organ dysfunction

The temporal course of S100B is stage-dependent and determines how dysfunction in one organ is transmitted to other organs (**Table 2**). In acute brain injury (trauma, cardiac surgery, stroke), the release is rapid and, with a ~25-min circulatory half-life, serum levels peak early and normalize within hours unless tissue damage and barrier disruption persist (26, 40); an analogous acute pattern is plausible for gut-derived S100B after enteric injury, although serial gut–portal measurements are lacking. In contrast, in chronic liver disease, serum S100B behaves as an accumulating marker; it increases with severity (bilirubin, creatinine, and MELD), is influenced by renal clearance and portosystemic shunting, and is associated with mortality rather than with the HE grade alone (25, 43). Thus, the same serum value can denote an acute glial event or a chronic multi-organ burden, and along the axis, the sequence is best read as a time-staggered cascade: gut injury raises portal S100B and microbial products; the liver, as the first-pass organ, develops fibrosis; and only with hepatic failure do ammonia, bile acids, cytokines, and S100B spill over systemically to reach the brain, where astrocytic autocrine S100B sustains neuroinflammation (7, 17, 24). Therefore, studies should report the kinetics (timing, duration, and rate of change) rather than single time-point values when S100B is used for cross-organ inference (**Table 2**).

## 8 S100B couples immune-cell infiltration with enteric glial immune–barrier regulation

Quantitative S100B–immune-infiltration correlations are emerging mainly in cancer and neuroinflammatory contexts and provide a hypothesis for the axis as a whole. In HCC, integrative (TIMER) analyses show S100B expression positively and significantly correlated with B-cell, CD8+ and CD4+ T-cell, macrophage, neutrophil and dendritic-cell infiltration; hypoxia upregulates S100B in HepG2 cells via HIF-1α binding to the S100B promoter; and S100B-correlated networks include chemokines, immune-checkpoint molecules and epithelial–mesenchymal-transition genes (41). Supporting observations exist in the healthy liver (S100B is largely expressed by immune cells such as natural killer cells) (41), brain (S100B/RAGE-driven neutrophil recruitment and NET formation after traumatic brain injury) (18, 29), and gut (S100B-dependent cytokine release recruiting neutrophils during Clostridioides difficile infection) (12). S100B, therefore, plausibly contributes to immune-cell recruitment and immunoregulation in every compartment of the axis, and serum S100B may partly mirror the immune-inflammatory burden. Direct per-tissue infiltration scoring against local S100B remains scarce and should be generated in prospective cohorts with spatial validation (**Table 2**).

The gut is a particularly informative compartment in which these immune-related functions converge on a defined glial population. EGCs are integral to the enteric nervous system and have historically been viewed as supportive cells for neurons (39). Current evidence indicates that they sense microbial signals, toxins, inflammatory cytokines, and neurotransmitters, and participate in motility, mucosal immunity, barrier repair, and modulation of visceral pain (44–46). Thus, EGCs can be considered a key cell population at the neuroimmune barrier interface of the brain–gut–liver axis (**Figure 2**). Consistent with the spatial map and kinetics discussed above, the checkpoint function of EGC-derived S100B is context- and concentration-dependent. Rhythmic colonic motor activity requires basal enteric-glial S100B signaling that is independent of RAGE (35), whereas sustained S100B elevation during inflammation shifts EGCs toward an inflammatory amplifier phenotype (12, 13).

**Figure 2:**
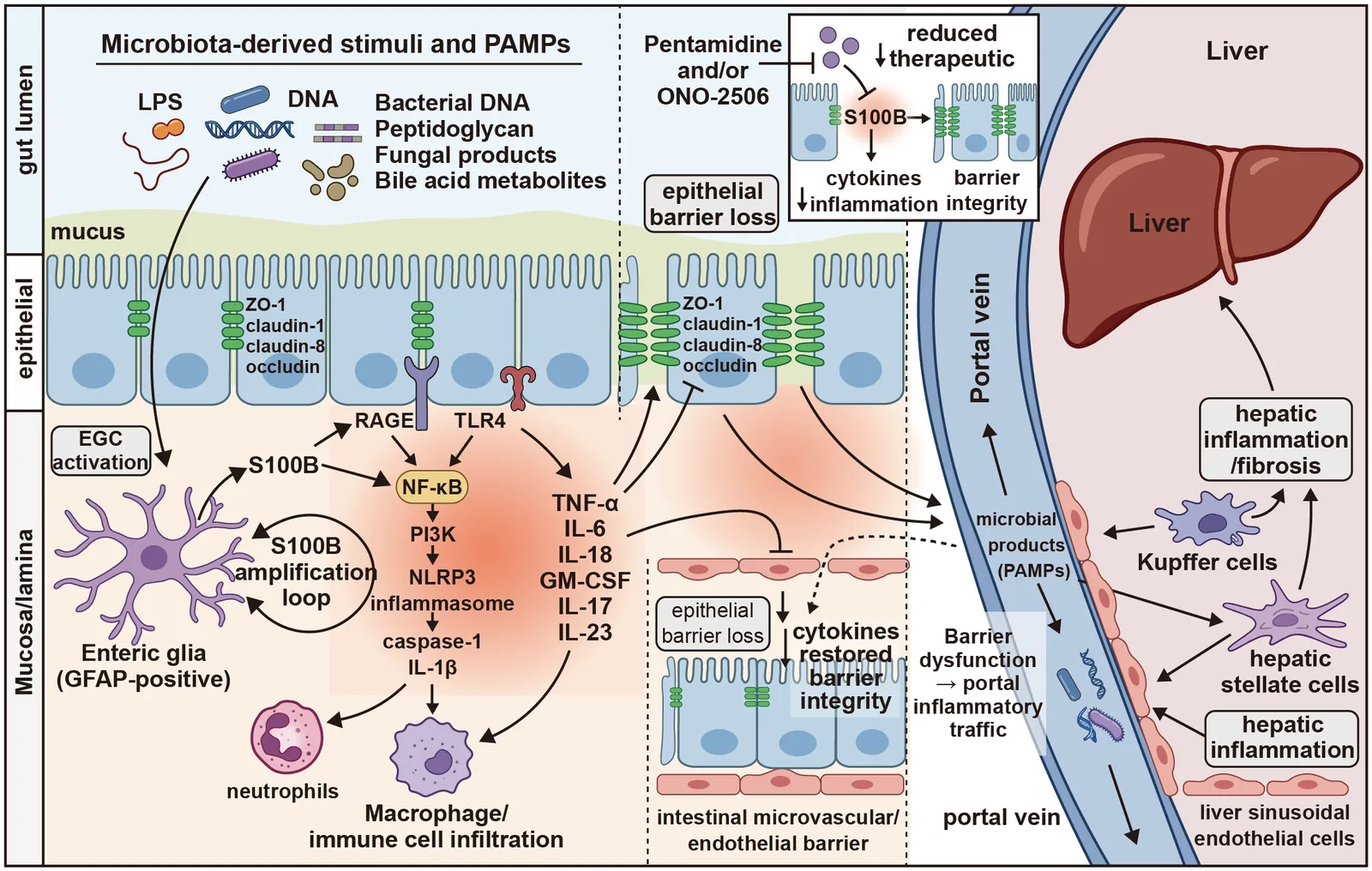
Enteric glial S100B regulates intestinal barrier and portal inflammation. Pathogen-associated molecular patterns (PAMPs) in the intestinal lumen activate EGCs in the lamina propria, promoting S100B release. S100B establishes a positive feedback loop and, through RAGE/TLR4–NF-κB–NLRP3 signaling, drives the production of multiple inflammatory cytokines, leading to disruption of the epithelial and microvascular endothelial barriers. Consequent barrier dysfunction facilitates the portal translocation of microbial products and inflammatory mediators, thereby activating hepatic Kupffer cells and HSCs and inducing hepatic inflammation and fibrosis. Targeted inhibition of S100B with pentamidine or ONO-2506 attenuates inflammation and restores barrier integrity.

Direct evidence of the pro-inflammatory role of S100B comes from infectious enteritis models. In Clostridioides difficile infections, colonic and fecal S100B levels are elevated in patients and infected mice, with higher fecal levels in those with diarrhea (12). In 5-fluorouracil-induced colitis, S100B/RAGE/NFκB expression increased (47); pentamidine, an S100B inhibitor, reduced IL1β, IL18, IL6, GMCSF, TNFα, IL17 and IL23, attenuated neutrophil recruitment and tissue injury (12), and decreased RAGE/NFκB expression (47). In EGCs, C. difficile toxins A and B induced IL6 expression through the S100B–RAGE/PI3K/NFκB axis (12). These findings indicate that S100B is not only a post-injury marker but also an effector molecule within an inflammatory amplification loop (**Figure 2**).

Research on inflammatory bowel disease (IBD) further supports the pathological relevance of the EGC–S100B axis. In a dextran sulfate sodium (DSS) colitis model, enteric glial A2B adenosine receptor signaling promoted barrier dysfunction, and glia-specific adenosine A2B receptor (A2BR) ablation restored the localization of tight junction proteins, including claudin-1, claudin-8 and occludin (46). A 2025 study published in ACS Nano showed that a colon-targeted oral nanosystem delivering pentamidine reduced EGC hyperactivation, S100B, and reactive oxygen species (ROS) and improved mucosal barrier integrity and immune homeostasis in ulcerative colitis (UC) mice (13). These observations suggest that EGC-derived S100B represents a druggable node at the intersection of neuroinflammation and immunoinflammation in IBD.

In diarrhea-predominant irritable bowel syndrome (IBS-D), S100B has been associated with microbial tryptophan metabolism. Patients showed reduced fecal indole metabolites, decreased AHR/cytochrome P450 family 1 subfamily A member 1 (CYP1A1) signaling and lower ZO-1 expression, alongside increased colonic mucosal S100B, NFκB and NLRP3; the S100B/AHR ratio correlated positively with symptom severity, NLRP3, NFκB and substance P expression (36). This supports a model in which reduced microbiota-derived indole-AHR signaling may weaken the restraint of enteric glial inflammation, thereby favoring S100B-associated low-grade inflammation, barrier impairment, and visceral sensitization (**Figure 2**). This human associative evidence, together with the S100B–immune-infiltration correlations presented above, suggests that EGC-derived S100B should be viewed as a quantitative checkpoint of mucosal immune tone rather than a qualitative on/off marker (13, 36, 41).

## 9 S100B shares a lineage with, but diverges functionally from, other S100 family members

The human S100 family comprises approximately 21 members that share a conserved EF-hand architecture but are not functionally interchangeable (37, 48). S100B is most closely related to S100A1, S100A2, and the S100A4 subgroup and behaves as a glial-specific, constitutively secreted protein with strong RAGE affinity (28, 37). In contrast, S100A8/A9 (calprotectin) and S100A12 are prototypical phagocyte-derived alarmins that are induced and actively released by neutrophils and monocytes during acute inflammation, act through TLR4 and RAGE, and are used clinically as biomarkers (e.g., fecal calprotectin in inflammatory bowel disease (IBD)) (42, 48). S100A4 is a pro-metastatic factor associated with epithelial–mesenchymal transition, whereas S100A11 and S100P are overexpressed in HCC and have been evaluated as diagnostic and prognostic markers, along with alpha-fetoprotein (37, 49). In healthy individuals, most S100 proteins are cell-specific and expressed at low levels, and in pathology, several are newly expressed in unusual cell types (e.g., hepatocyte S100A8/A9 during inflammation-related carcinogenesis) (37). The practical implications are that “S100” must never be treated as a single entity (assays are isoform-specific), and that within the axis S100B interpretation should be integrated with S100A8/A9 (intestinal and systemic inflammation) and with hepatic S100A11/S100P signatures when liver cancer is the clinical question (**Table 1**) (48, 49).

## 10 S100B links intestinal barrier dysfunction to portal inflammatory traffic

The intestinal barrier comprises the mucus layer, epithelial tight junctions, immune defenses, glial regulation, and intestinal vascular barrier. Barrier breakdown allows lipopolysaccharide (LPS), bacterial deoxyribonucleic acid (DNA), peptidoglycans, fungal products, bile acid metabolites, and other pathogen-associated molecular patterns (PAMPs) to enter the portal circulation, where they activate Kupffer cells, HSCs, and liver sinusoidal endothelial cells, driving hepatic inflammation and fibrosis (8, 50–52).

S100B may affect portal inflammatory load via three routes. First, EGC-derived S100B can directly weaken both the epithelial and vascular barriers. In HFD-fed mice, 1,25(OH)_2_D_3_ suppressed EGC S100B release by increasing colonic butyrate production and reducing colonic hyperpermeability; the S100B pathway inhibitor ONO-2506 similarly ameliorated HFD-induced barrier injury (14). Secondly, S100B can synergize with LPS/TLR4, RAGE, and NLRP3 signaling to amplify local inflammation. HFD induced excessive EGC NLRP3/caspase-1/IL-1β activation, which compromised the epithelial barrier; NLRP3 deficiency or IL-1β receptor blockade attenuated this damage (53). Third, because S100B itself can cross vascular barriers and is detectable systemically, gut-derived S100B is a plausible portal- and systemic-vectored signal, in addition to the co-release of PAMPs. Direct measurement of portal versus systemic S100B in patients would clarify this route (26, 40).

In the context of liver disease, the relevance of intestinal barrier disruption lies in the conversion of local intestinal inflammation into portal and hepatic inflammation (**Figure 2**). MASLD, ALD, and viral hepatitis are associated with dysbiosis, reduced barrier function, and altered enteric metabolites (54–56). Therefore, even when direct causal evidence for S100B is lacking in certain liver diseases, S100B remains a plausible candidate molecule at the EGC barrier–portal inflammation interface.

## 11 S100B and liver diseases

### 11.1 S100B drives cholestatic liver fibrosis

Cholestatic fibrosis is a disease for which intrahepatic S100B activity is most directly supported by experimental evidence. In bile- duct-ligated mice, S100B was mainly localized to biliary epithelial cells and increased with ductular reaction and fibrosis progression. RAGE was also elevated and co-localized with S100B in proliferating cholangiocytes and activated HSCs (24). *In vitro*, recombinant S100B dose-dependently upregulated fibrotic markers such as α-SMA and Col1a1 in HSC-T6 cells, whereas RAGE siRNA suppressed S100B-induced HSC activation (24).

These data point to a potential “cholangiocyte S100B–RAGE–HSC” paracrine amplification loop (**Figure 3**). During cholestatic injury, cholangiocytes express S100B, which promotes ductular reactions, HSC activation, and collagen deposition upon RAGE engagement. This loop may operate alongside TGF-β, bile acid receptors, inflammatory cytokines and extracellular matrix (ECM) remodeling to determine the progression of portal fibrosis. Within the fibrosis–cirrhosis continuum, this represents the earliest experimentally verified S100B-dependent stage and is therefore the most defensible therapeutic window (24, 49).

**Figure 3:**
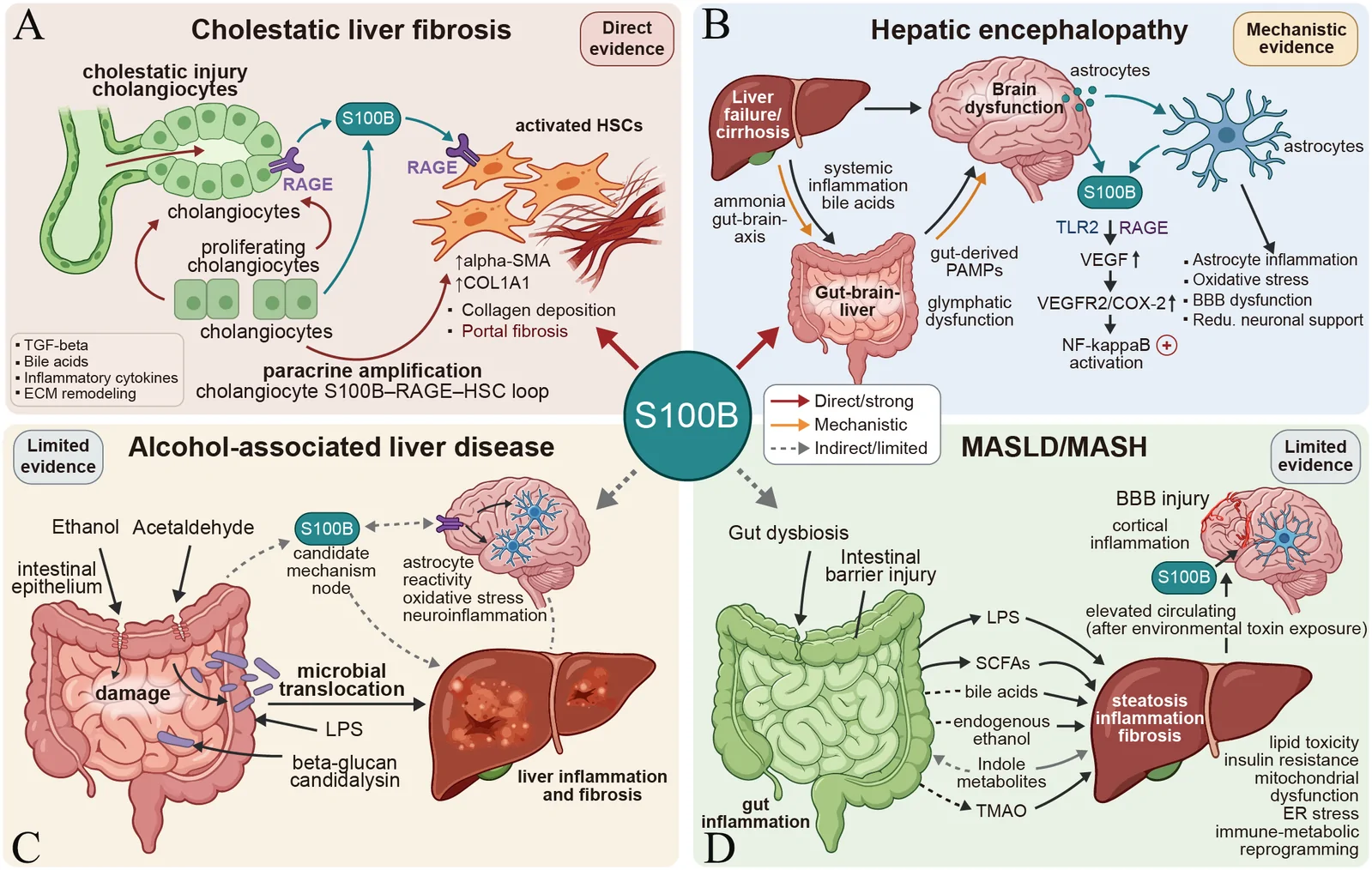
Evidence hierarchy of S100B in liver disease pathogenesis. **(A)** Cholestatic liver fibrosis: S100B engages RAGE on cholangiocytes and HSCs, forming a paracrine amplification loop that promotes HSC activation and collagen deposition, directly driving fibrosis. **(B)** Hepatic encephalopathy: liver dysfunction releases inflammatory mediators via the gut–liver–brain axis, promoting astrocytic S100B release, which activates RAGE/NF-κB signaling, thereby mediating BBB disruption and impairing neuronal support. **(C)** Alcohol-associated liver disease: ethanol/acetaldehyde disrupt the intestinal barrier, triggering hepatic inflammation and astrocytic stress; S100B may serve as a mechanistic node. **(D)** MASLD/MASH: dysbiosis and barrier injury facilitate metabolite transport to the liver, inducing lipotoxicity and metabolic inflammation. Direct clinical evidence for S100B in MASLD/MASH is limited; its elevation is often associated with systemic inflammation and exposure to environmental toxins.

### 11.2 S100B is linked to MASLD-related hepatic and neuroinflammatory phenotypes

MASLD is a prototypical metabolic liver disease linked to dysfunction of the gut–liver axis. Current models implicate lipotoxicity, insulin resistance, mitochondrial dysfunction, endoplasmic reticulum (ER) stress, immunometabolic reprogramming, dysbiosis, and barrier injury in its pathogenesis (54, 57–59). Gut microbiota and their metabolites, including LPS, short-chain fatty acids, bile acids, endogenous ethanol, indole derivatives, and trimethylamine N-oxide (TMAO), influence hepatic inflammation, lipid metabolism, and fibrosis (8, 54, 60). Beyond steatosis and steatohepatitis, progression toward cirrhosis and HCC is accompanied by characteristic S100-family signatures (S100A4, S100A6, S100A8/A9, S100A11, and S100P) that are stage-dependent and measurable in serum, although S100B itself has not yet been validated at any MASLD stage (49). Direct clinical evidence for the role of S100B in MASLD remains limited, but animal studies have suggested its involvement in MASLD-associated neuroinflammation and BBB abnormalities. Nonalcoholic fatty liver disease (NAFLD) mice exposed to microcystin-LR developed cortical inflammation, reduced BBB tight junction proteins, and neurodegenerative changes, accompanied by elevated circulating S100B (61); NLRP3-dependent inflammation is linked to BBB injury, and S100B may participate in astrocyte–neuron damage signaling (61). This supports a model linking metabolic liver disease to systemic inflammation, BBB dysfunction, and astrocytic S100B (**Figure 3**) but does not establish S100B as a major driver of MASLD-related brain alterations.

### 11.3 S100B is a plausible but unproven node in alcohol-associated liver disease

In ALD, ethanol and acetaldehyde damage the intestinal epithelium, disrupt tight junctions, alter bacterial and fungal communities, and increase portal influx of LPS, β-glucan, candidalysin and other microbial products, thereby inducing hepatic inflammation and fibrosis (55, 62, 63). ALD also involves brain reward circuitry, mood disorders, cognitive impairment, and neuroinflammation (9, 64), making it a classic gut–liver–brain axis disease (**Figure 3**). However, direct evidence for the role of S100B in ALD is scarce. One study reported no significant S100B elevation in healthy volunteers with a stable blood alcohol level of 100 mg/dL, whereas intoxicated patients with much higher alcohol levels showed elevated S100B (65); However, that study did not assess liver injury. S100B remains a plausible but unproven candidate for ALD, potentially linking alcohol-induced gut-barrier injury to astrocytic responses. The interpretation of serum S100B levels in patients with ALD is further complicated by malnutrition, inflammation, cirrhosis, and variability in renal function. Like MASLD, ALD spans the same fibrosis–cirrhosis–HCC continuum, and the probable translation of alcohol-induced intestinal and hepatic injury into glial S100B responses is best tested by serial sampling of the intestinal, portal, and systemic compartments (54, 63, 65).

### 11.4 S100B is only indirectly implicated in viral hepatitis

Hepatitis B virus (HBV) and hepatitis C virus (HCV) infections affect the gut–liver axis through chronic inflammation, immune activation, altered bile acid metabolism and dysbiosis (3, 66). In HBV, microbial metabolites such as short-chain fatty acids, bile acids, tryptophan metabolites, TMAO, vitamins, and hydrogen sulfide may influence disease progression and prognosis (56, 67). In HCV, a sustained virological response partially restores the gut microbiota and fecal bile acid profiles, and an increase in *Blautia* is correlated with improved fibrosis and liver function (68). *In vitro* and replicon studies have shown that S100 proteins, including S100B, bind to FKBP8/FKBP38 and inhibit FKBP8–Hsp90 and FKBP8–NS5A interactions, and that overexpression suppresses HCV replication (69). This suggests a potential host restriction mechanism; however, these findings require *in vivo* validation. In a cohort of 64 patients with chronic hepatitis B/C receiving interferon, serum S100B and brain-derived neurotrophic factor (BDNF) levels decreased significantly by week two, coinciding with rising depression and anxiety scores (70). This suggests that treatment-related immune and neurotrophic changes may confound peripheral S100B measurements. Therefore, the treatment regimen, sampling timing, and neuropsychiatric status should be considered when interpreting serum S100B levels in patients with chronic hepatitis.

Currently, there is insufficient evidence supporting the direct regulation of S100B by viral hepatitis. A more cautious interpretation is that viral hepatitis may affect S100B-related networks indirectly through chronic inflammation, disturbances of the gut–liver axis, progression to cirrhosis, and increased HE risk, rather than through direct viral-driven S100B expression.

## 12 The S100B and S100-family signature tracks the progression from cirrhosis to hepatocellular carcinoma

Liver disease is best understood as a continuum, in which fibrosis, cirrhosis, and HCC represent successive stages with distinct biological contexts and, critically, distinct S100B evidence (10, 49). At the fibrosis stage, direct experimental data supported the presence of a cholangiocyte–HSC S100B/RAGE loop (24). In the cirrhosis stage, serum S100B is elevated, correlates with MELD, bilirubin, and creatinine levels, and predicts 1-year mortality, consistent with a multi-organ inflammatory burden rather than a liver-specific signal (25). Direct clinical data on serum S100B levels at the HCC stage are scarce; however, two complementary lines of evidence have emerged. First, the broader S100 family is a recognized feature of the metabolic-liver-to-HCC continuum: S100A4, S100A6, S100A8/A9, S100A11, and S100P are overexpressed in steatohepatitis and HCC tissue and/or serum and are correlated with tumor stage, recurrence, and survival (49). Second, transcriptomic analyses specifically implicate S100B in the hypoxic, immune microenvironment of HCC: HIF-1α transcriptionally upregulates S100B in HepG2 cells, S100B knockdown attenuates hypoxia-induced invasion and migration, and S100B expression correlates with immune cell infiltration and immune checkpoint networks (41). These stage-specific patterns reinforce the central message of this review: S100B and the S100 family as a whole should be interpreted as a disease- and context-dependent readout rather than as a single disease biomarker (**Table 3**) (25, 41, 49).

**Table 3:** Disease-specific evidence and mechanistic interpretation of S100B-related brain-gut-liver regulation.

Disease context	Main axis disturbance	S100B-related evidence	Mechanistic interpretation	Strength and caveat	References
Clostridioides difficile infection	Toxin-induced enteric glial activation and mucosal inflammation	Colonic and fecal S100B increased; pentamidine reduced cytokines and tissue injury	S100B-RAGE/PI3K/NF-κB amplifies EGC inflammatory response	Strong original evidence, mainly preclinical plus patient samples	(12)
Ulcerative colitis	EGC hyperactivation, ROS, barrier injury	Colon-targeted pentamidine nanosystem reduced S100B, ROS, cytokines and restored barrier	S100B-centered inflammatory loop in EGCs	Strong preclinical therapeutic evidence	(13)
IBS-D	Low-grade inflammation, altered tryptophan-indole metabolism, visceral sensation	S100B/AHR ratio correlated with IBS-SSS, NLRP3, NF-κB, and substance P	Reduced indole-AHR signaling may release EGC inflammatory tone	Human associative evidence; causality unresolved	(36)
Diet-induced obesity/MASLD-related gut dysfunction	HFD-induced gliosis, NLRP3 activation and barrier leak	EGC NLRP3/caspase-1/IL-1β worsened epithelial barrier injury	Metabolic inflammation primes EGC-barrier axis	Strong preclinical evidence but S100B not always central	(53)
Cholestatic liver fibrosis	Ductular reaction and HSC activation	S100B/RAGE upregulated in BDL liver; S100B activated HSC via RAGE	Cholangiocyte-S100B-RAGE-HSC fibrogenic loop	Strong direct liver evidence; human validation lacking	(24)
MASLD/NAFLD brain comorbidity	Metabolic inflammation, BBB dysfunction, neuroinflammation	NAFLD plus toxin exposure increased circulating S100B and BBB/neuroinflammatory injury	S100B may reflect astrocyte-BBB response in metabolic liver disease	Mechanistic but second-hit model; not generalizable to all MASLD	(61)
Alcohol-associated liver disease	Alcohol-induced dysbiosis, gut leak, systemic and CNS inflammation	Direct S100B data limited; gut-liver-brain framework strong	S100B is plausible but unproven glial readout	Requires ALD-specific tissue and longitudinal studies	(9, 55)
Viral hepatitis	Chronic inflammation, microbiota and bile acid disturbance	Direct S100B evidence insufficient	S100B likely relevant mainly after cirrhosis/HE develops	Avoid overstatement; indirect evidence only	(56, 68)
Hepatic encephalopathy	Ammonia, inflammation, BBB/NGVU dysfunction	MHE astrocytes show S100B autocrine inflammatory loop; serum studies conflicting	S100B may be both effector and composite biomarker	Strong mechanistic evidence; clinical specificity limited	(17, 25, 43)

## 13 S100B connects ammonia, inflammation and neurogliovascular dysfunction in hepatic encephalopathy

Histological findings in HE, including tissue staining, exemplified the multiorgan interactions of the brain–gut–liver axis (**Figure 3**). The traditional model emphasizes the conversion of ammonia to glutamine in astrocytes by glutamine synthetase, which induces osmotic stress and astrocyte swelling. More recent frameworks define HE as a multifactorial condition involving ammonia, systemic inflammation, bile acids, gut-derived PAMPs, BBB dysfunction, microglial activation, neurogliovascular unit (NGVU) impairment and glymphatic clearance deficits (7, 71, 72).

S100B has dual significance in mechanistic- and biomarker-related contexts. Mechanistically, in a minimal HE (MHE)-like rat model, cerebral S100B expression and autocrine release increased; S100B promoted VEGF expression via TLR2 and RAGE, which in turn activated NF-κB through VEGFR2/COX-2 crosstalk, induced astrocytic inflammation and oxidative stress, and impaired neuronal support (17). This study advanced S100B from a mere “astrocyte injury marker” to an “astrocyte inflammatory amplifier” and placed the molecular events precisely at the neurogliovascular unit, where ammonia-driven astrocytic stress meets neuroinflammation (17, 71).

However, the clinical utility of serum S100B as a marker remains unclear. A 2023 study of 95 cirrhotic patients found that serum S100B did not effectively distinguish among no HE, MHE, and overt HE, but correlated with age, bilirubin, creatinine, and MELD score; S100B ≥ 35 pg/mL combined with MELD ≥ 13 predicted 1-year mortality (25). Conversely, a 2025 small-sample study reported that serum S100B correlated with Psychometric Hepatic Encephalopathy Score (PHES) performance, and 83% of patients with MHE had S100B levels above the healthy reference range (43). Together with the kinetic framework described above, these divergent findings indicate that the interpretation of S100B requires time-resolved sampling and adjustment for age/renal function, rather than a single cut-off (25, 26, 43). These findings are not necessarily contradictory; they suggest that S100B reflects a composite measure of glial reactivity, barrier damage, and disease burden rather than being an HE-specific marker.

## 14 S100B extends its relevance to neurological and gastrointestinal diseases beyond the liver

S100B is widely used as a marker of astrocyte activation, BBB disruption, and neuroinflammation in neurological disorders. Parkinson’s disease, epilepsy, traumatic brain injury, delirium, amyotrophic lateral sclerosis (ALS), and chronic pain/anxiety models have implicated S100B/RAGE signaling or S100B-related glial responses in neuroinflammation and neurovascular unit injury (18, 34, 73–76). These observations have implications for hepatology: elevated S100B levels in chronic liver disease should not be automatically attributed to HE but may also reflect cerebrovascular disease, metabolic disturbances, infection, surgical stress, renal impairment, or other neurological comorbidities.

Gastrointestinal diseases, including IBD, irritable bowel syndrome (IBS), infectious enteritis, nonsteroidal anti-inflammatory drug (NSAID) enteropathy, metabolic barrier injury, EGC responses, and S100B release, have been documented (12, 36, 45, 46, 77). In the dinitrobenzene sulfonic acid (DNBS) acute colitis model, intestinal inflammation was accompanied by the upregulation of transient receptor potential vanilloid 1 (TRPV1) and S100B in the colonic myenteric plexus, dorsal root ganglia, and periaqueductal gray, suggesting that peripheral EGC responses may influence central sensitization along pain pathways (45). This provides mechanistic clues for S100B involvement in the “enteritis–visceral pain–brain response” axis. These observations, together with the alarmin function and immune cell correlates discussed above, indicate that S100B-dependent signaling is shared across organ systems and must therefore be interpreted in the context of the full clinical picture (organ of origin, kinetics, and concurrent comorbid conditions) (12, 18, 29, 45).

## 15 Integrated regulatory mechanisms converge on glial–barrier–immune loops

The evidence reviewed above indicates the presence of several interconnected regulatory loops. First, gut-derived stimuli (dysbiosis, toxins, HFD, alcohol) activate EGCs to release S100B, which, via RAGE/TLR4/NF-κB/NLRP3 signaling, promotes the production of inflammatory cytokines and downregulates barrier proteins (ZO-1, occludin, claudin-1), thereby increasing epithelial and vascular permeability (12–14, 53). This allows LPS and other PAMPs to reach the liver via portal circulation, thereby activating Kupffer cells and HSCs. Second, in cholestatic injury, cholangiocyte-derived S100B engages RAGE on HSCs to promote fibrogenic markers including α-SMA, Col1a1 and TIMP-1 (24), providing a mechanistic link between ductular reactions and portal fibrosis. Third, cirrhosis, portosystemic shunting, and liver failure allow ammonia, bile acids, inflammatory cytokines, and microbial products to enter systemic circulation. Ammonia primarily affects astrocyte metabolism, whereas systemic inflammation and bile acids affect BBB, microglia, and neurogliovascular units (7, 72, 78). Astrocytic autocrine S100B drives inflammation and oxidative stress through TLR2/RAGE–VEGF–VEGFR2/COX-2–NF-κB signaling (17). Fourth, microbiota metabolites such as butyrate and indole derivatives modulate this network: butyrate suppresses EGC S100B release (14), whereas reduced indole–AHR signaling correlates with elevated S100B and NLRP3/NF-κB activation (36). Superimposed on these circuits, the kinetic and spatial principles developed earlier predict that the same loops propagate as a time-staggered cascade (gut first, portal liver, and systemic brain), which can be exploited experimentally by sequentially sampling the portal and systemic compartments (7, 17, 40). Therefore, future interventions should consider microbiota metabolite remodeling rather than solely focusing on S100B inhibition.

The strengths and limitations of S100B-related evidence across disease contexts are summarized in **Table 3** to facilitate the understanding of mechanistic targets and intervention directions.

## 16 Therapeutic implications require stage- and axis-specific strategies

Therapeutic strategies targeting S100B must consider its context-dependent functions. Low concentrations may support neuronal survival and tissue repair (30); therefore, long-term systemic blockade could carry risks, particularly given the constitutive trophic secretion of S100B by healthy glia and its alarmin duality (28, 29). Disease- and axis-specific stratification is a more rational approach. When gut-derived inflammation predominates, targeting EGC S100B and barrier repair may be prioritized; for cholestatic fibrosis, the S100B/RAGE–HSC axis may be explored; and in HE, combination strategies addressing ammonia, inflammation, microbiota, BBB integrity, and glial responses are warranted (**Figure 4**). In HCC, the emerging immunomodulatory role of S100B prevents broad inhibition and points toward an immune context-aware evaluation (37, 41).

**Figure 4:**
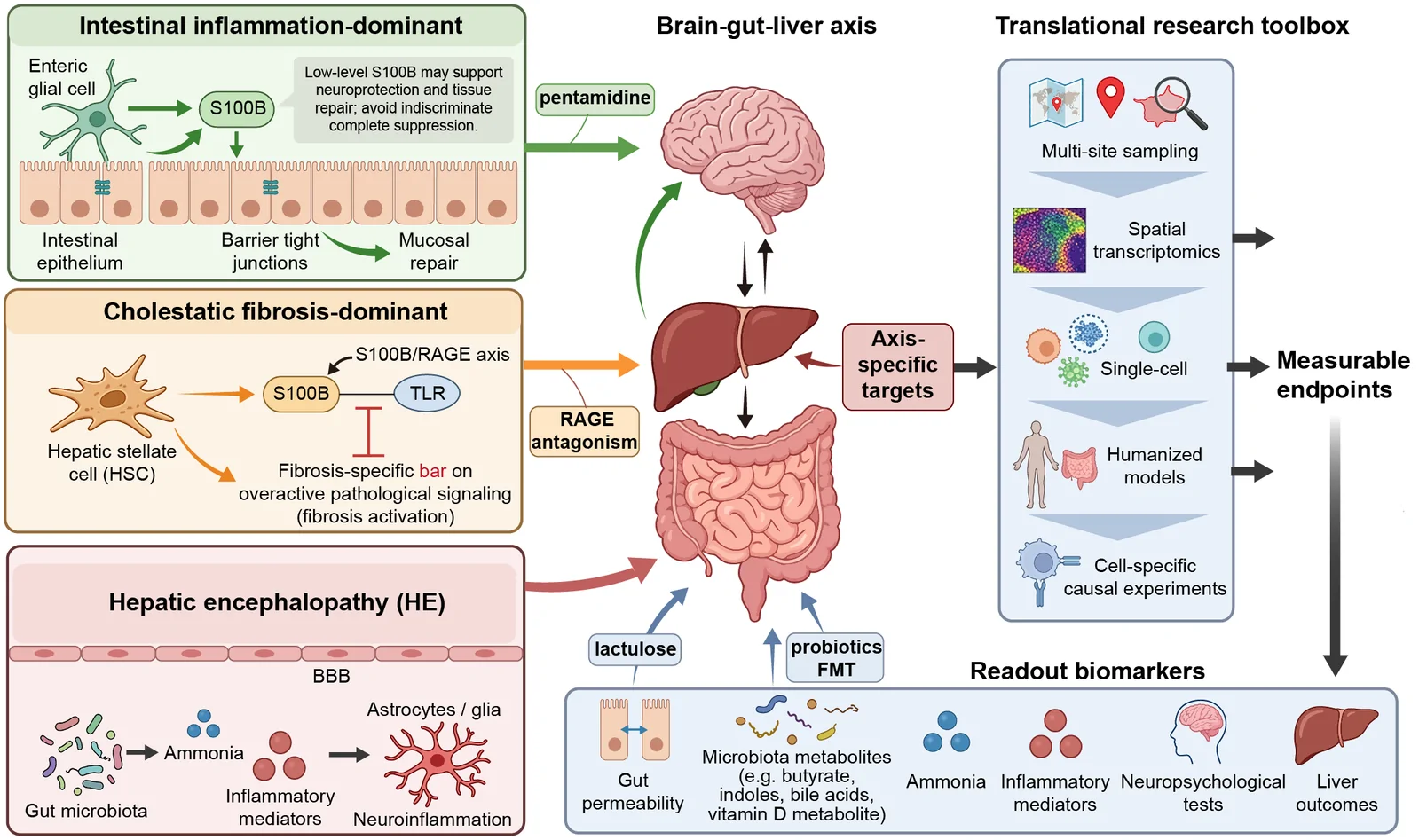
Translational roadmap for S100B-targeted brain-gut-liver research. Future studies should integrate multi-site sampling, spatial and single-cell methods, humanized models, and cell-specific causal experiments. S100B should be evaluated together with gut permeability, microbiota metabolites, ammonia, inflammatory mediators, neuropsychological tests and liver outcomes.

Preclinical studies have shown that S100B pathway inhibitors, such as pentamidine and ONO-2506, attenuate injury in Clostridioides difficile infection (CDI), UC, HFD-related barrier dysfunction, and neuroinflammatory models (12–14, 76). RAGE antagonism also shows promise in reducing S100B/RAGE/NET-mediated secondary inflammation after traumatic brain injury (18, 32). However, because the RAGE and TLR pathways are involved in host defense and tissue repair, long-term systemic blockade requires careful safety evaluation and therapeutic windows should be aligned with the kinetic profiles described above (acute high-release bursts versus chronic low-grade spillover) (26, 40).

Microbiota metabolite modulation offers an alternative approach aligned with the biology of the brain–gut–liver axis. The 1,25(OH)_2_D_3_–butyrate–S100B axis, indole–AHR–S100B pathway, bile acid receptor signaling, and probiotic/fecal microbiota transplantation (FMT) strategies may indirectly modulate S100B-related networks (14, 68, 79, 80). Current treatments remain focused on reducing gut-derived ammonia and systemic inflammation (e.g., lactulose, rifaximin, probiotics, and FMT), and S100B is better suited as a future composite mechanistic stratifier rather than an immediate independent therapeutic target.

## 17 Discussion

Extrapolating these findings between rodents and humans requires caution at several levels. The S100B protein is highly conserved across mammals, and the astrocytic/enteric-glial expression pattern is shared, which is why mechanistic models (bile duct ligation, DSS/5-FU colitis, MHE rats, and traumatic brain injury) are considered representative of the corresponding human processes (17, 24). Nevertheless, there were important quantitative differences. Relative to body size, the human brain contributes to a much larger glial cell mass, and human serum S100B is additionally influenced by extracerebral sources (adipose tissue and muscle), renal function, age, and body mass, whereas rodent sera behave more homogeneously under standardized conditions (26, 40). Species differences in RAGE signaling kinetics and assay standardization (antibody specificity and reference ranges) further complicate direct translation (11, 26). In the liver, the rodent bile duct ligation model provides the strongest evidence for the cholangiocyte–HSC S100B/RAGE loop, but human cholestatic cohorts have not yet been examined for intrahepatic S100B/RAGE co-expression. Conversely, the S100B–immune-infiltration association in HCC was derived from human transcriptomic data and a human cell line, illustrating how human and rodent evidence complement each other (24, 41). Therefore, emerging mechanistic findings should be confirmed in human tissues (e.g., spatial transcriptomics and multiplexed imaging of liver and gut biopsies) before anti-S100B strategies are proposed in patients.

Several consensus points emerged. S100B is an important indicator of glial activation and barrier injury. S100B reflects the transition from a homeostatic-supportive state to a reactive or inflammatory state in astrocytes and EGCs (12, 17, 26). Second, sustained elevation of extracellular S100B promotes inflammatory amplification via RAGE/TLR/NF-κB pathways in models of CDI, UC, MHE, and traumatic brain injury (TBI) (12, 13, 17, 18). Third, the S100B/RAGE axis has been experimentally linked to cholestatic fibrosis, with S100B upregulation in cholangiocytes and HSCs promoting the expression of fibrotic markers (24). Fourth, HE integrates gut-derived ammonia, systemic inflammation, bile acids, and BBB dysfunction, with S100B positioned at the intersection of these processes (7, 17, 71). Another point arising from this synthesis is that S100B biology is spatial and temporal; its functions can only be interpreted with knowledge of the producing cell, disease stage, and release and clearance kinetics (**Table 3**) (25, 29, 40, 41).

However, important limitations of this study remain. First, the diagnostic utility of serum S100B for MHE or HE is uncertain. Although some studies have reported a correlation with PHES (43), larger studies found that it did not distinguish HE severity and was confounded by age, bilirubin level, creatinine level, and MELD (25). Thus, S100B is best used as part of a multimarker model rather than as a stand-alone HE biomarker. Second, S100B effector functions have been demonstrated in CDI, UC, BDL, and MHE models (12, 13, 17, 24), whereas human serum studies suggest that S100B likely reflects the multiorgan disease burden (25–27). Third, S100B signaling extends beyond RAGE to include TLR2/TLR4, VEGF/VEGFR2, ADAM10/β-catenin, and metabolic axes (17, 31, 36). Fourth, the therapeutic window for S100B is narrow; low levels may support tissue repair, whereas long-term non-selective inhibition could be harmful (26, 81). Fifth, direct evidence linking S100B to immune cell infiltration is largely derived from transcriptomic and one-cell line studies in HCC, and per-tissue spatial validation in the gut and brain is required (41).

Methodologically, most mechanistic evidence is derived from animal models, cell experiments, or small clinical studies, and human longitudinal causal data are scarce. The models of CDI, DSS colitis, HFD, BDL, hyperammonemia, TBI, and acute liver failure represent distinct pathological stressors that cannot be used interchangeably. Direct evidence for S100B in MASLD, ALD, and viral hepatitis remains limited and is largely inferred from the gut–liver and liver–brain axes. Future studies should employ cell-specific genetic models (e.g., EGC- or astrocyte-specific S100B knockout and cholangiocyte- or HSC-specific RAGE knockout) and standardize S100B detection methods, cutoff values, and multimarker diagnostic models to avoid overinterpreting a single serum marker as organ- or disease-specific. Studies should record sampling kinetics (acute versus chronic, repeated measures), biological age, renal function, and systemic inflammation, and should ideally measure S100B in the serum, CSF/feces, and local tissue in parallel (**Table 3**) (26, 40).

## 18 Conclusion and future perspectives

S100B functions as an integrating molecule at the glial–barrier–immune interface of the brain–gut–liver axis but is currently best understood as a candidate mechanistic node and composite biomarker rather than a disease-specific indicator. The available evidence supports three main points: (i) EGC-derived S100B contributes to intestinal inflammatory amplification and barrier injury, with direct evidence in CDI and UC models (12, 13); (ii) the S100B/RAGE axis promotes cholestatic liver fibrosis, linking ductular reactions, HSC activation and ECM deposition (24); and (iii) astrocytic S100B autocrine signaling is implicated in MHE/HE-related neuroinflammation and oxidative stress (17). However, clinical interpretation of serum S100B is confounded by tissue origin, BBB integrity, liver disease severity, renal function, age and systemic inflammation (25–27). A further point is that S100B acts within a spatial and temporal framework: alarmin function, immune-cell correlates, tissue-specific expression and stage-dependent kinetics together define whether an elevation is a local event or a systemic network signal (29, 40, 41, 49).

Future studies should prioritize source identification, causal validation, and stratified modeling. Multi-site synchronous sampling (serum, feces, CSF, intestinal mucosa, liver tissue, and portal blood) across well-characterized cohorts would help dissect tissue-specific contributions of S100B. Single-cell ribonucleic acid (RNA) sequencing, spatial transcriptomics, and metabolomics should be integrated to characterize the S100B-positive cell populations and their local microenvironments. Human organoid and organ-on-chip models (enteric glia–epithelial, gut–liver, and BBB) may enable the mechanistic dissection of trans-barrier S100B signaling (**Figure 4**). In chronic liver disease cohorts, S100B should be analyzed alongside MELD, ammonia, bile acids, inflammatory cytokines, gut barrier markers, microbial metabolites, and neuropsychological tests, rather than in isolation. Therapeutic strategies should avoid complete S100B inhibition in favor of combination approaches—microbiota metabolite remodeling, barrier repair, RAGE/TLR modulation, ammonia reduction, and BBB protection—tailored to the disease stage and predominant axial involvement.

Overall, S100B offers a valuable entry point for understanding the links between glial responses, barrier disruption, hepatic inflammation, and neuroinflammation within the brain–gut–liver axis. However, current evidence does not support its use as an independent diagnostic or therapeutic target. With further clarification of its tissue origin, receptor pathways, disease-stage specificity, and clinical endpoints, S100B may emerge as a useful candidate for mechanistic stratification and the development of interventions in brain–gut–liver axis disorders.
